# The effects of changing meteorological parameters on fatal aortic catastrophes

**DOI:** 10.1186/s12872-020-01575-1

**Published:** 2020-06-13

**Authors:** Brigitta Szilágyi, Márton Berczeli, Attila Lovas, Zoltán Oláh, Klára Törő, Péter Sótonyi

**Affiliations:** 1 Institute of Mathematics, Budapest University of Technology and Economics, 3 Műegyetem rakpart, Budapest, 1111 Hungary; 2 Heart and Vascular Center, Department of Vascular Surgery, Semmelweis University, 68 Városmajor street, Budapest, 1122 Hungary; 3 Department of Forensic Medicine, Semmelweis University, 93 Üllői road, Budapest, 1093 Hungary

**Keywords:** Acute aortic dissection, Ruptured aortic aneurysm, Cox process model, Non-parametric approach, Statistical learning

## Abstract

**Background:**

Over the span of the last decade, medical research has been increasingly putting greater emphasis on the study of meteorological parameters due to their connection to cardiovascular diseases. The main goal of this study was to explore the relationship between fatal aortic catastrophes and changes in atmospheric pressure and temperature.

**Methods:**

We used a Cox process model to quantify the effects of environmental factors on sudden deaths resulting from aortic catastrophes. We used transfer entropy to draw conclusion about the causal connection between mortality and meteorological parameters. Our main tool was a computer program which we developed earlier in order to evaluate the relationship between pulmonary embolism mortality and weather on data sets comprised of aortic aneurysm (AA) and acute aortic dissection (AAD) cases, where one of these two medical conditions had led to fatal rupture of the aorta. Our source for these cases were the autopsy databases of Semmelweis University, from the time period of 1994 to 2014. We have examined 160 aneurysm and 130 dissection cases in relation to changes in meteorological parameters. The algorythm implemented in our program is based on a non-parametric a Cox process model. It is capable of splitting slowly varying unknown global trends from fluctuations potentially caused by weather. Furthermore, it allows us to explore complex non-linear interactions between meteorological parameters and mortality.

**Results:**

Model measures the relative growth of the expected number of events on the n^th^ day caused by the deviation of environmental parameters from its mean value. The connection between ruptured aortic aneurysms (rAA) and changes in atmospheric pressure is more significant than their connection with mean daily temperatures. With an increase in atmospheric pressure, the rate of rAA mortality also increased. The effects of meteorological parameters were weaker for deaths resulting from acute aortic dissections (AAD), although low mean daily temperatures increased the intensity of occurrence for AAD-related deaths.

**Conclusion:**

The occurrence rate of fatal aortic catastrophes showed a slight dependence on the two examined parameters within our groups.

## Background

Acute aortic syndrome (AAS) is a condition that can develop suddenly in any section of the aorta: the thorax, the abdomen or even both. Its cause is most often either acute aortic dissection (AAD) or ruptured aortic aneurysm (rAA). Its importance is key, as AAS mortality caused by rAA and AD can exceed 80% [1]. It is well established for both of these medical conditions that hypertension and smoking constitute a significant risk factor, which greatly increase the incidence rate of AAS [2–4].

The effects of changing weather conditions on blood pressure have already been studied [5–7]. Increased mortality rate for cardio- and cerebrovascular patients under extreme weather conditions (extreme cold and heat) is also known [8, 9]. Several articles have been published on this subject, although many controversies exist as to whether a correlation is present [10–16]. We think the controversy stems from the differences in local meteorological conditions, observed parameters and methodologies, the combination of which leads to different conclusions. In addition, it is recognized that the analysis of a correlation does not give information about the etiology.

Several studies discuss the connection between cardiovascular diseases (pulmonary embolism, ruptured aortic aneurysm, acute coronary syndrome, aortic dissection) and changes in meteorological parameters [10, 17, 18]. A large amount of articles examine the effects of weather on aneurysms and dissections [10–16]. All of these strive to prove a connection between the given medical condition and the meteorological parameter or its changes.

In the past decades, the Generalized Additive Model (GAM) became a popular analytical tool in epidemiology, especially in studies on the effects of environmental variables on public health [19]. However, these methods suffer from serious limitations such as the presence of confounding variables. Furthermore, by construction, complex non-linear interactions between explicative variables are ruled out. We use a robust non-parametric alternative which is based on a Cox process model, where the non-parametric intensity is the product of a multidimensional link function and a slowly varying hidden trend. The non-parametric nature of the model enables it to learn complex trends that parametric models could not determine. Furthermore, we do not need any prior information about the joint distribution of the weather parameters. Our research group had earlier developed a computer program based on such a model for studying the connection between pulmonary embolism and meteorological conditions [18]. In our recent study, we performed calculations with this program on the data set of fatal aorta aneurysms and aortic dissections.

## Methods

### Forensic data

For our study we reviewed 20 years of autopsy data from the Department of Forensic and Insurance Medicine of Semmelweis University. In this Institute autopsies are performed on persons who died either in the public spaces of Budapest, in ambulance cars or in a hospital within 24 h following the primarily performed surgical procedure. The Department performed a total of 17,760 autopsies between the ^1st^ of January 1994 and the ^1st^ of January 2014. We analyzed every case where the leading diagnosis or cause of death was either aortic aneurysm or aortic dissection. We selected these based on the International Classification of Diseases, Tenth Revision, Clinical Modification (ICD-10-CM) codes (Table 1). Out of the selected cases, after a detailed review of the autopsy reports, we have retrieved 290 cases where the following conditions were met: 1. the sole cause of death was confirmed to be an aortic rupture caused by AA or AD 2. the time and circumstances of the aortic rupture could be determined with great accuracy.

**Table 1:** The International Classification of Diseases, Tenth Revision, Clinical Modification (ICD-10-CM) codes

I7100	Dissection of unspecified site of aorta
I7101	Dissection of thoracic aorta
I7102	Dissection of abdominal aorta
I7103	Dissection of thoracoabdominal aorta
I712	Thoracic aortic aneurysm, without rupture
I713	Abdominal aortic aneurysm, rupture
I714	Abdominal aortic aneurysm, without rupture
I715	Thoracoabdominal aortic aneurysm, rupture
I716	Thoracoabdominal aortic aneurysm, without rupture
I718	Aortic aneurysm of unspecified site, ruptured
I719	Aortic aneurysm of unspecified site, without rupture

### Meteorological data

We have examined clinical data of patients who died in AAS and compared these to the mean daily temperature on the day of death and the change in atmospheric pressure between the day of death and the day before. For this, we used data of meteorological parameters available from the database of the European Centre for Medium-Range Weather Forecast (ECMWF). All data from ECMWF are region specific for Pest County, where the events happened.

Meteorologists have begun to calculate mean daily temperatures in 1966, using the average of 4 daily measurements taken at 0, 6, 12, and 18 UTC (Coordinated Universal Time). In Europe, during the daylight-saving period, Central-European Time (CET, UTC + 1), and in the summer Central-European Summer Time (CEST, UTC + 2) is used. Therefore, measurements are taken at 1, 7, 13, and 19 UTC in the winter and 2, 8, 14, and 20 UTC in the summer. If these four measurements were unavailable for any given date, we used the average of the highest and lowest measured daily temperature.

Average atmospheric pressure in Hungary is around 1016.5 hPa (sea-level adjusted pressure).

### Statistical data analysis

Statistical analysis of data can be divided into different phases. First we inferred causation between environmental factors and mortality using transfer entropy (TE) and determined time delay between cause and effect. Let *N*_*t*_ be the registered number of events on the *t*^*th*^ day and *X*_*t*_ ∈ *R*^*p*^ the actual value of the weather parameters, where *p* denotes the number of influencing factors taken into account. Transfer entropy *TE*_*X* → *N*_ measures the uncertainty in future values of *N*_*t*_ by knowing the past values of *X*_*t*_ given the past values of *N*_*t*_ [20].Unlike Pearson correlation, TE measures causation between exposure and disease. For vector auto-regressive processes, it reduces to Granger causality. We defined the optimal time delay between *X*_*t*_ and *N*_*t*_ as the lag value *τ* ∈ *N* for which ${TE}_{X^{\tau}\to N}$ attains its first local maximum, where *X*^*τ*^ stands for the time series *X*_*t*_ lagged by *τ*. Since no parametric distribution of errors is known for transfer entropy, we used surrogate data to test the statistical significance of values obtained for TE. A more detailed description of this approach with applications in neuroscience can be found in Vicente et al., 2011 [21]. Let ${\overset{\sim }{X}}^{\tau }$ be the random permutation of *X*^*τ*^. Obviously, shuffling destroys causal relationships and thus the quantity $\varDelta {TE}_{X^{\tau}\to N}={TE}_{X^{\tau}\to N}-{TE}_{{\overset{\sim }{X}}^{\tau}\to N}$ has positive expectation whenever *X*_*t* − *τ*_ causes *N*_*t*_. We stated the following null and alternative hypotheses.

$$ {\mathrm{H}}_0:\varDelta {TE}_{X^t\to N}=0\kern0.5em \mathrm{vs}.\kern0.5em {\mathrm{H}}_1:\varDelta {TE}_{X^{t\kern1em }\to N}>0. $$

We employed one-sided *t*-test to determine the maximal *p*-value at which the null hypothesis can be rejected.

The second phase is about the quantification of potentially existing causal relationships between weather and AACs. We assume that the conditional distribution of *N*_*t*_ given *X*_*t*_ is Poisson with intensity parameter *λ*(*t*, *X*_*t*_) which is exactly the expected number of events on the *t*^*th*^ day, provided that the environmental factors take value *X*_*t*_. We also assume that the intensity parameter can be written as a product in which the dependence of *λ* on *t* and *x* is separated, that is: *λ*(*t*, *X*) = *f*(*t*)^2^*g*(*X*). Here *g* : *R*^*p*^ → [0, ∞) describes the effect of *X*_*t*_ on *N*_*t*_ and *f* : *N* → [0, ∞) captures the global hidden trend which we cannot observe directly. Note that, unlike in other commonly used models, we have no restriction for the functional form of *f* and *g*. We implemented a generalized EM algorithm to infer these functions from data. Our algorithm minimizes the utility function

$$ {U}_{N,X,g}(f)={\sum}_{t=1}^T{f}_t^2g\left({X}_t\right)-{N}_t\mathit{\log}\left({f}_t^2g\left({X}_t\right)\right)+\beta {\sum}_{t=1}^T{\left({f}_t-{f}_{t-1}\right)}^2, $$

where *f*_1_ − *f*_0_ ≔ 0, 0 *log* (0) is defined to be zero and the regularization parameter *β* > 0 is responsible to avoid over-fitting. The utility function is essentially the negative log-likelihood function plus a regularization term that controls the complexity of *f*. Let $\underset{\_}{X}$ be the time average of *X*_*t*_ over the observation period and define the hidden trend as $g\left(\underset{\_}{X}\right){f}_t^2$ and introduce the risk measure $r(x)=\left(\frac{g(X)}{g\left(\underset{\_}{X}\right)}-1\right)\times 100\%$ that is the relative percentage growth of *λ*(*t*, *x*).

## Results

Over the 290 selected cases, the cause of death was ruptured aneurysm in 160 cases and aortic dissection in 130 cases. In the rAA group, the average age was 70.26 years, while in the AAD group, it was 62.17 years. In the first group the male/female ratio was 1.96: 1.00 (106: 54), in the second it was 1.71: 1.00 (82: 48). In the rAA group the peak of occurrences appeared to be in the winter, while in the AD group, it was in the spring. We did not report a statistically significant relationship neither in seasonal variation nor in the monthly distribution of the events. Table 2 and Table 3 shows the seasonal and monthly variation of the events.

**Table 2:** The seasonal variation of the events

*N* = 290	rAA (*n* = 160)	AAD (*n* = 130)	Sum
Spring	38	39	77
Summer	39	28	67
Autumn	38	27	65
Winter	45	36	81
Sum	160	130	290

**Table 3:** The monthly variation of the events

*N* = 290	rAA (*n* = 160)	rAD (*n* = 130)
January	15	14
February	14	12
March	13	15
April	16	10
May	9	14
June	14	8
July	13	10
August	12	10
September	14	9
October	10	7
November	14	11
December	16	10

Results of our causal analysis are summarized in Table 4. It can be stated that the transfer entropy between the timeseries of registered events and lagged climate data reaches its maximum when the lag value is set to one, which can be interpreted as that the weather on the preceding day has the greatest influence on the incidence of rAA and AAD. The only exception is rAA, where the air pressure changes between the 4th and 3rd day before the event.

**Table 4:** Optimal time lag between cause and effect and related *p*-values

	*T*_*avg*_[°*C*]	Δ*P*[*hPa*]
rAA	*τ* = 1	*τ* = 3
	*p* = 0.61	*p* = 0.75
AAD	*τ* = 1	*τ* = 1
	*p* = 0.28	*p* = 0.66

Low p-values indicate a weak causal relationship. Between aortic dissection and mean daily temperature TE is not statistically significant, but values obtained in all other combinations are.

**Fig. 1:**
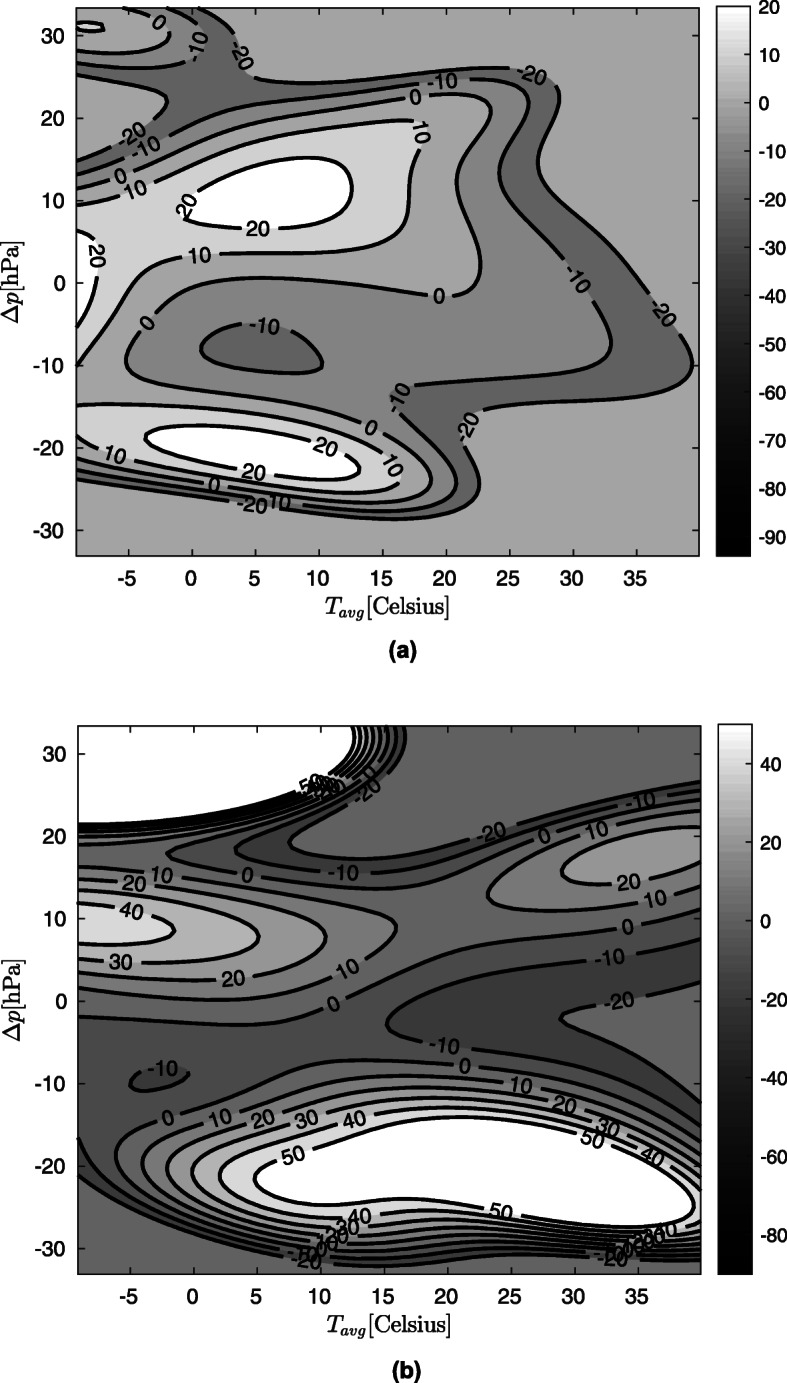
**a** Relative occurrence rates for rAA by mean daily temperature and atmospheric pressure changes. **b**: Relative occurrence rates for AAD by mean daily temperature and atmospheric pressure changes

Figure 1a and b show the relative occurrence rate for rAA and AAD,calculated from the data.

**Fig. 2:**
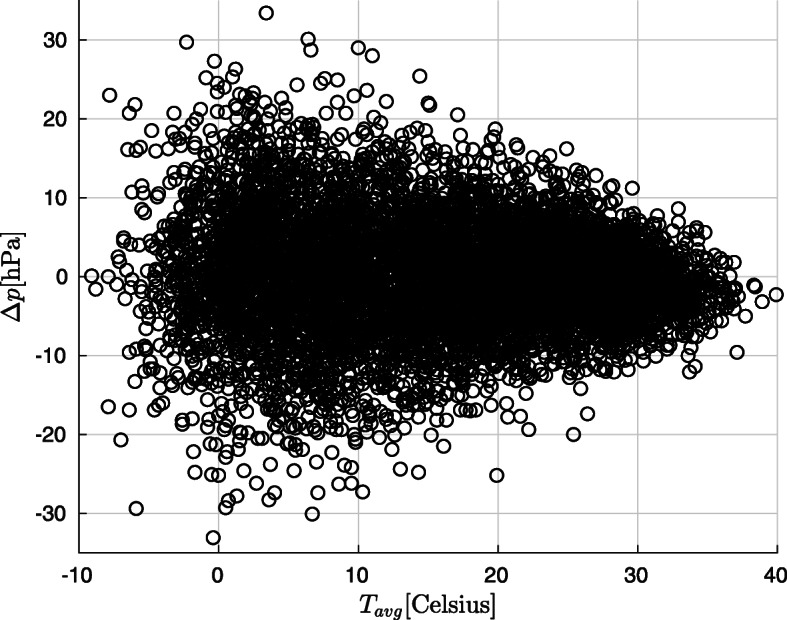
Daily mean temperature and air pressure change values recorded during the observation period

In Fig. 1a and b, the rAA and AAD mortality *r* values are shown as a function of the mean temperature measured on the day before the event and the change in air pressure relative to the day before. Knowledge of possible values of weather parameters will aid interpretation: Fig. 2 shows daily mean temperature and air pressure change values recorded during the observation period.

Figure 1a and b show the expected percentage change in the daily occurrence of rAA and AAD as a function of daily average temperature and atmospheric air pressure change in correlation to the previous day. For instance, we can observe that if the daily average temperature were less than 10 °C, then 5 hPa increase in daily air pressure change would result in an at least 10% growth of the incidence of rAA and AAD.

The significance of *r* values ​​in Fig. 1 a and b at a given point increase with the density of the point cloud. That is, the more measurements we have around the given point, the better we can predict the occurrence of rAA or AAD. Based on this, the following conclusions can be drawn:

- At rAA, in cold weather (mean temperature 0 °C to 10 °C), the occurrence of the disease is increased by a growth in air pressure and decreased by a decline thereof. The figure shows that this could mean up to 20% increase or 10% decrease.
- The incidence of AAD is at most slightly dependent on temperature. Here too, cold weather and elevated air pressure are a risk factor, but the effect is weaker than at rAA.
- In both figures, extreme values must be ignored because there are not enough measurement in these areas (see Fig. 2).

**Fig. 3:**
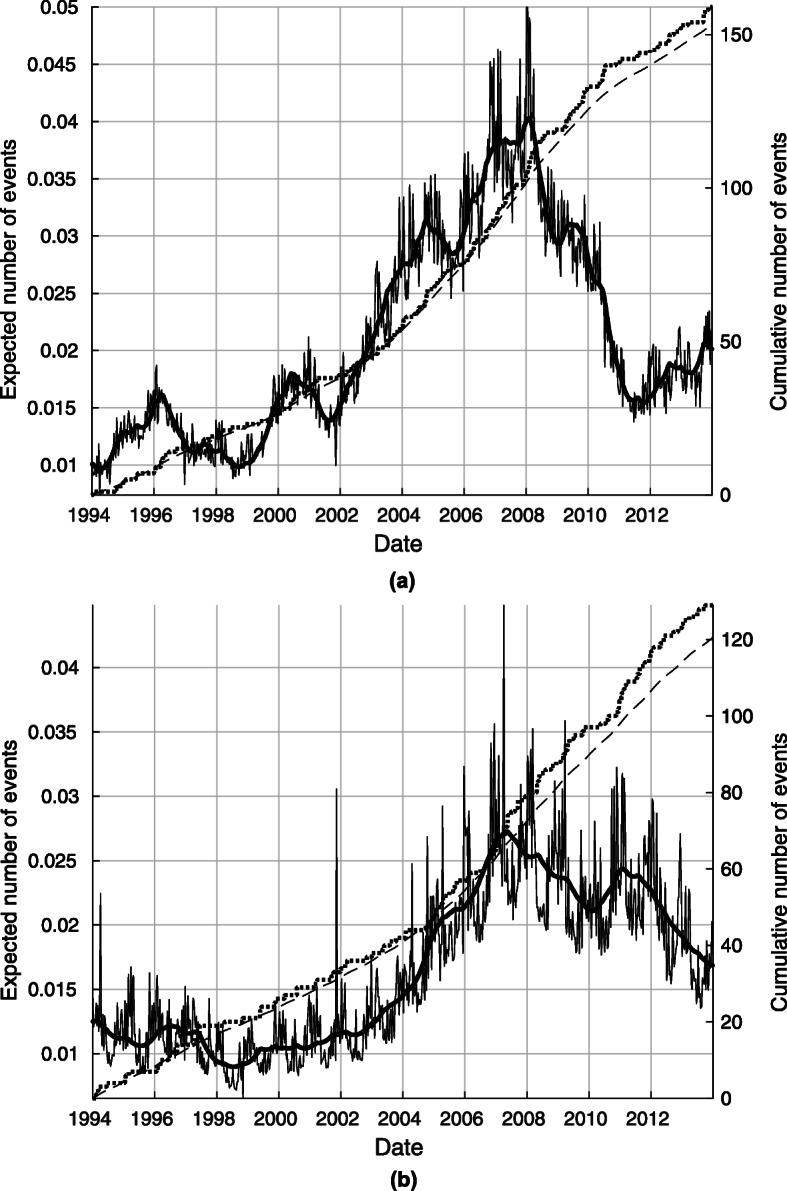
**a** The real and cumulative event numbers for rAA. **b**: The real and cumulative event numbers for AAD

Figure 3a and b depicts the real and model cumulative event numbers. The appropriateness of the model is shown by the fact that the values it predicts are well in line with reality, with a difference of less than 10 persons in 20 years.

Estimated intensities and cumulative trends are presented in Fig. 3, where on the left axis, the solid line means estimated daily intensity of rAA and AAD modulated by weather conditions and the thick line stands for the directly unobservable hidden trend which can be traced back to relatively slow changes in the population such as migration, aging, changing habits, etc. The appropriateness of the model is shown by the fact that it can learn complex non-parametric hidden trends from data, moreover by that splitting the trend that we can infer any complex nonlinear functional relationship between the response variable and the explicative variables. In Fig. 3, we present also the cumulative number of events too (right axis), where the solid line denotes the estimated number of events and the dotted line represents the actual number of rAA and AAD cases up to a given day.

Also shown in Fig. 3 is the number of events in a day predicted by the model, indicated by a thin solid line. The global trend that is not directly observable is represented by a thick line. In both diseases, an abnormal peak in the trend is observed over the 2002–2012 period. It is important to note that the statistical model used allows us to decouple the influence of weather from the background trend.

## Discussion

In our retrospective study we focussed on the connection of AAC occurrence and meteorological parameters. Using a new methodological approach, we aimed to achieve clinically more significant results that may help vascular surgeons work in treating AACs.

The connections between meteorological parameters and acute aortic catastrophes have previously been studied previously [10–13, 22]. Early studies focussed primarily on seasonal and monthly changes in mortality rate [10, 11, 14, 23, 24].

Our research team studied cases of both dissection and ruptured aneurysm. Including two decades worth of data in our study is unique in terms of time range. Available literature shows that on average, studies cover around 6–17 years, while a longer time range offers a better chance at observing trends [12, 22].

We have examined 160 AA and 130 AD cases and in comparison to the previous studies listed above, our numbers are not outstanding. Liapis and al. observed 226 cases of abdominal aortic aneurysms [11]. Ballaro and colleagues covered a significantly larger number of cases however, their study did not extend to changes in atmospheric pressure as their focus was on seasonal changes in incidence [10]. Bown and colleagues examined 580 cases of ruptured aortic aneurysm [22]. Upshur and colleagues included 2373 cases of acute aortic catastrophe in their study, without distinguishing between aneurysms and dissections [14]. Comparing these numbers, we can state that higher event numbers give more reliable results, in light of which our work is limited due to smaller sample size and lack of accurate timing information.

In our study the frequency of events reaches its peak in winter (rAA) and in spring (AAD). We we compared this to others’ results and found that Liapis et al. from Greece observed an autumnal peak, Ballaro and Bown from the UK found a winter peak, as did Upshur et al. from Canada, while Majd et al. from Germany also reported an autumnal peak. According to a meta-analysis and systematic review on the topic rAA, seasonal variation exists with higher incidence in autumn and winter [13]. Takagi et al. concluded form a meta-analysis and systematic review concluded that AAD also shows seasonal variation with a winter peak [25].

According to Wu et al., rAA and mean daily or other atmospheric pressure or other atmospheric pressure does not have significant impact on occurrence. Controversy between studies arise from the different climate patterns and methodology used.

Our model suggests that when mean daily temperatures are above 25 °C, the incidence of ruptures will be significantly lower, and will not significantly depend on changes in atmospheric pressure. Although observations reveal that many events occur on warm days with stable atmospheric pressure, our model predicts a low level of intensity for these days. The reason for this inconsistency is that there are many such days in a year, and, as a result, more events are likely to occur on them, even with a low intensity rate, than on days with higher intensity rates, but under rarer meteorological conditions.

Low *p*-values indicate a weak causal relationship. The reason for this is that the transfer entropy requires more sample for accurate estimation. Our sample size might not be sufficient for this methodology, which may be the reason why we obtained a lower *p* value than usual (*p* > 0.95) in epidemiological practice. We also need to consider that the accidental confusion of *X* elements does not necessarily destroy every possible causal relationship, thus the $\Delta  {TE}_{X^{\tau}\to N}$ statistic distorts in a negative direction.

Our model provides valuable information on the relationship between illness and weather on each day. There is no need to exclude days from the analysis like it was done by Petitti et al., to reduce seasonal effects [26].

Our research lacks the pathophysiological background and the direct connection between the occurrence and meteorological parameters. We believe that the mechanism of action could be the combination of sympathic activity change, arterial spasm, ambient blood pressure and the hematological characteristic change of platelets and red blood cells [13, 27–30].

Having presented several earlier papers on the subject, it can be seen that the overwhelming majority of our results are based on a novel method, and therefore the placement and comparison of these results with previous approaches is dubious.

## Conclusion

After analyzing 20 years’ worth of forensic autopsy data, we conclude that changes in atmospheric pressure have a more significant effect on the incidence rate of fatal rAA cases than mean daily temperature. Sudden rise in atmospheric pressure can increase the intensity of mortality resulting from AA. The effect of these two meteorological parameters is likely to be lower for fatal AAD cases. The intensity of AD-related mortality can be connected to mean daily temperatures, where cold favors the occurrence of ruptures resulting from AD.

Through the application of this model, we are able to analyze the connection between other medical conditions and weather or a completely different independent variable. This opens up a series of opportunities both in the practice of public health care and in other areas.

## Data Availability

All data and material available at Semmelweis University, Budapest, Hungary. Data could be requested from Prof. Péter Sótonyi M.D.,Ph.D. The meteorological data was gathered from the database of the European Centre for Medium-Range Weather Forecast (ECMWF). Administrative permission is not required to access the data.

## Abbreviations

AAC: Acute aortic catastrophes
rAA: Ruptured aortic aneurysm
rAAA: Ruptured aortic abdominal aneurysm
AAD: Acute aortic dissection
CEST: Central-European summer time
UTC: Coordinated universal time
ECMWF: European centre for medium-range weather forecast
ICD-10-CM: International classification of diseases, tenth revision, clinical modification
